# Highly beneficial outcome in severe acute necrotizing encephalopathy with tocilizumab treatment

**DOI:** 10.1007/s00415-024-12661-1

**Published:** 2024-09-06

**Authors:** Alexander Balck, Lara M. Lange, Alexander Neumann, Georg Royl, Philipp Jung, Jens Schaumberg, Norbert Brüggemann, Philipp J. Koch

**Affiliations:** 1 Department of Neurology, University of Lübeck and University Hospital Schleswig-Holstein, Campus Lübeck, Ratzeburger Allee 160, 23562 Lübeck, Germany; 2 Department of Neuroradiology, University of Lübeck and University Hospital Schleswig-Holstein, Campus Lübeck, Lübeck, Germany; 3 Department of Paediatrics, University of Lübeck and University Hospital Schleswig-Holstein, Campus Lübeck, Lübeck, Germany; 4 Department of Neurology, SANA Clinic Lübeck, Lübeck, Germany

## Background

Acute necrotizing encephalopathy (ANE) is a severe neurologic condition that can arise following various systemic infections, including influenza and SARS-CoV-2 [1]. The outcome of ANE ranges from complete recovery (< 10%) to recovery of the acute episode with persistent deficits to death, with a mortality rate of up to 30% [2]. Affected individuals are, in most cases, young and present with rapid changes in consciousness, focal neurologic deficits, and epileptic seizures. Neuroimaging typically reveals symmetric, bilateral deep-gray matter lesions, often involving the thalami, with evidence of necrosis and/or hemorrhage [1].

In contrast to typical infectious diseases, brain damage is not directly caused by the infectious agent but by the accompanying ‘cytokine storm,’ including interferons, interleukins, and chemokines, caused by systemic infection [3]. As expected, early steroid treatment was demonstrated to result in a better clinical outcome for children without brainstem lesions [4].

Tocilizumab is an Interleukin 6 (IL-6) inhibitor that was initially developed to treat long-term autoimmune disorders such as rheumatoid arthritis. However, its clinical usage was soon expanded to treat diseases with acute severe autoimmune reactions, such as cytokine release syndromes following systemic infections, e.g., with SARS-CoV-2 [5]. In ANE, high levels of IL-6 correlate with worse clinical outcomes and are thought to affect the blood–brain barrier, leading to neurotoxicity and cytotoxic edema. Only a limited number of ANE patients have been treated with Tocilizumab, but the reported short- and long-term outcomes were favorable [6, 7].

Here, we report a 17-year-old female (index patient) and her mother with ANE following influenza A infection and gastroenteritis, respectively. The index patient was successfully treated with tocilizumab in addition to standard therapy with steroids and intravenous immunoglobulin therapy (IVIG); her mother experienced full recovery after high doses of i.v. Methylprednisolone.

## Case reports

**Fig. 1:**
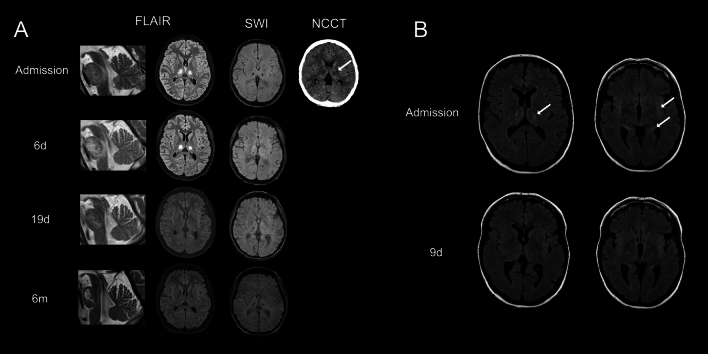
**|** Imaging at admission and follow-ups of the index patient and her mother. **A** Cerebral signal alterations of the index patient are shown at onset and their development over three consecutive follow-up measurements 6 days, 19 days, and 6 months after admission. Non-contrast cranial CT (NCCT) at admission showed bilateral symmetric signal alterations, mainly in the pons and thalami. Hyperintensities in Fluid-attenuated inversion recovery (FLAIR) were visible in the medulla oblongata, pons, and bilaterally in the cerebellum, thalami, hippocampi, and external/extreme capsule with a symmetric distribution as well as high mainly precentral cortical alterations. Those FLAIR hyperintensities were in part also present with b1000 diffusion restrictions, ADC hypointensities (not shown), and signal alterations in susceptibility-weighted imaging (SWI). The follow-up MRIs showed overall regression of FLAIR lesions, diffusion restrictions, and SWI abnormalities. At the same time, a mild contrast agent enhancement in the right thalamus was still visible at 19 days post-admission (not shown). **B** FLAIR images at admission and 9 days follow-up of the mother show bilateral hyperintensities in the thalamus, external/extreme capsule, and parahippocampal gyrus at onset, which were almost entirely reversible over time

The index patient was a 17-year-old female patient who was admitted to the emergency department with a coma as the leading symptom. According to her mother, she had felt sick since the previous evening. The Glasgow Coma Scale was three on admission, so she was intubated for protection. In addition to the comatose state, the neurologic exam revealed equal-sized, light-reactive pupils with bilateral positive Babinski signs. The initial cranial CT and MRI scan showed bilateral, symmetric signal alterations in the thalami, hippocampi, and external capsules and brainstem (Fig. 1A). An influenza A antigen test was positive. Cerebrospinal fluid (CSF) showed no pleocytosis, intrathecal antibody synthesis, or oligoclonal bands but an elevated protein level (703 mg/l). The serum IL-6 level was 156 ng/l (reference < 7). CSF analyses regarding autoantibodies causing autoimmune encephalitis were negative, including Amphiphysin, CASPR 2, GABA B, LGI 1, Ma2, NMDA, AMPA, Hu, GAD, MOG, and Neuropil antibodies.

A diagnosis of ANE was suspected due to the combination of coma, the typical MRI pattern, positive influenza testing, and isolated elevation of CSF protein. The microbiological workup was negative for differential diagnoses, including SARS-CoV2, HIV, HSV 1/2, VZV, listeria, toxoplasmosis, and fungi. With an ANE-S Score of 5/9 (Brain-stem lesions, > 48 months of age, elevated CSF-protein), the patient was classified as high risk for unfavorable outcomes in ANE. For a detailed description of the scoring system, please see Yamamoto et al. [8]. Thus, treatment with 600 mg of tocilizumab was initiated at intervals of 5.5, 9, and 16 h after admission. In addition, we applied methylprednisolone (1000 mg i.v. for 5 days) and oseltamivir (75 mg for 10 days). Body temperature was kept normothermic and did not exceed 37 °C in the first 72 h. After discontinuation of sedation, there was no adequate waking response but minimal oral movement. An EEG showed severe general slowing. Two days after admission, the patient was in a locked-in state, being able to communicate via vertical eye movements when her eyelids were held open (Video 1).

Five days after admission, serum IL-6 level had decreased to 46.5 ng/l (reference < 7).

Six days after admission, the patient could open her eyes and communicate by nodding and head-shaking, extending and closing her fingers and toes. A follow-up MRI showed little progression of the pontine lesions but regression of all other lesions (Fig. 1A). Because of the progressive pontine lesions, we started additional treatment with 150 g IVIG for 5 days, which was followed by further continuous clinical improvement. After the endotracheal tube was removed 10 days after admission, she had aphonia.

At the time of discharge 18 days after submission, the patient was severely hypophonic, showed psychomotor slowing, and displayed mild tetra paresis and mild limb ataxia (modified Rankin score 4). There were no pyramidal signs or sensory deficits, and reflexes were normal.

Neurologic examination at a follow-up visit 6 months after initial admission revealed insignificant hemiataxia and mild but clinically irrelevant dysarthria (Video 1). A follow-up MRI showed minimal residual signal alterations bithalamically and in the pons (Fig. 1A). The patient reported no limitations in daily life but suffered from anxiety and mild depressive symptoms.

The mother of the index patient was hospitalized six months after her daughter with acute onset of nausea, vomiting, and diarrhea accompanied by severe disorientation and working memory deficits. The mother suffered a similar episode of gastroenteritis as a trigger for rapidly developing memory deficits about a year before, from which she made a prolonged full recovery without any specific therapy. MRI at onset showed symmetric signal alterations mainly affecting the thalamus, external capsule, and parahippocampal gyrus, similar to those of the index patient but sparing the pons (Fig. 1B). Next to elevated protein levels in the cerebrospinal fluid (640 mg/l), further blood examinations regarding autoimmune or infectious diseases were negative. Staging examinations did not show any suspect tumor. The ANE-S Score was 3/9. The patient was treated with methylprednisolone 1000 mg i.v. over five consecutive days and subsequent therapy with 60 mg p.o. Within days, the patient almost fully recovered with persistent working memory deficits for two weeks.

Single-gene testing performed on the index patient and her mother revealed no pathogenic variants in the *RANBP2* gene*.*

## Discussion

In ANE, a viral infection (Influenza, SARS-CoV2) triggers a cytokine storm, increasing susceptibility to oxidative stress and neurotoxicity [2]. ANE has been reported in 3.5–5% of influenza cases, with less than 10% experiencing full recovery and a mortality rate of 30%, highlighting the urgency for improved diagnostic methods and more effective treatments. Increased levels of IL-6 at an early stage of the disease have been repeatedly reported and are associated with poor clinical outcomes, suggesting a pivotal role in the pathological cascade [9–11]. Depending on viral contact, genetic polymorphism in promoter regions might promote a dysregulated immune response. Higher levels of IL-6, as well as TNFalpha, lower levels of IL-10, and alterations of further pro-inflammatory cytokines, are thought to induce endothelial cell injury and vascular inflammation, increasing blood–brain barrier and vascular permeability leading to neurotoxicity, cytotoxic edema, and neuronal and glial apoptosis [11, 12].

Consequently, the IL-6 inhibitor Tocilizumab, combined with corticosteroid therapy, has been introduced as a treatment with high potential for acute encephalopathy. However, thus far, only a few pediatric cases of ANE receiving Tocilizumab have been reported [6, 7, 13, 14]. The authors reported a favorable outcome despite severe ANE-S scores [7]. The current FDA guidelines approve the Off-label use of tocilizumab in cytokine release syndrome. Severe adverse events during therapy may occur and include upper respiratory infection, herpesvirus infections, severe allergic reactions, peripheral edema, diarrhea, and blood-cell depletion [15]. This documented patient with severe ANE enables us to expand upon existing evidence, suggesting that early administration of Tocilizumab in conjunction with standard corticosteroid and IVIG therapy, can yield substantial clinical enhancements, even in cases with brainstem involvement (as indicated by an ANE-S score of 5/9) and in older, adolescent patients. The application in mildly affected patients should be decided individually considering the patient’s clinical manifestation and the initial course of the disease. It remains an open question whether the observed brain lesions or high IL-6 levels independent of the clinical severity may justify an off-label use in selected patients. The rarity of the disease remains a challenge for the identification of treatment and outcome biomarkers, as systematic clinical studies will most likely not be possible in the near future.

To our knowledge, there is no evidence for alternative treatments such as plasmapheresis or antivirals [4, 16]. The efficacy of IVIG therapy or high dosage i.v. glucocorticoids, also in combination with tocilizumab, is not yet confirmed due to a limited number of case series. However, it is  likely to be effective and beneficial with a manageable risk [4, 17, 18]. Further add-on symptomatic treatment suggestions to improve overall outcome include hypothermia, reduction of intracranial pressure, and prevention of further organ failure [6, 13].

Sporadic ANE is usually induced by environmental factors, including pathogenic microorganisms such as influenza, herpes- or coronavirus, or drug-induced, including NSAIDs [12]. The presented case of recurrent ANE in an elderly female patient and a severe case of ANE in the patient's daughter is highly suggestive of genetic susceptibility. Missense mutations in the *RANBP2* have been discovered for family-segregated recurrent ANE with autosomal dominant traits and ~ 40% penetrance (ANE1 or ADANE) [19]. Its crucial intracellular role and interactions with mitochondria metabolism and nuclear signaling are believed to cause cytokine storms and metabolic and mitochondrial dysfunctions and to increase vulnerability to oxidative stress [2]. Further, genetic polymorphisms in IL-10, HLA-DR, and -DQ have been identified to contribute to the pathogenesis of ANE [12]. The role of polymorphisms in promotor regions of IL-6 is still under debate [11]. Therefore, performing genetic analyses in cases of ANE is crucial to understanding the underlying pathogenesis, developing novel treatment strategies, informing patients and relatives about the disease, and potentially testing family members. The absence of a *RANBP2* mutation in this family suggests there may be additional genetic causes of ANE.

Although this is only a case report and the effect of tocilizumab is thus not proven in controlled, randomized clinical trials, the illustrated case supports the usage of tocilizumab in severe ANE after risk–benefit evaluation.

Therefore, we recommend (i) anti-inflammatory treatment plus additional acute IL-6 blockade in severe manifestations, (ii) genetic testing for all new patients diagnosed with ANE, and, in case of a positive genetic finding, further testing of yet asymptomatic family members to stratify the risk for future ANE, (iii) apply preventive measures, including vaccinations for COVID-19 and influenza to avoid infections, for patients who suffered from ANE before and at-risk family members.

## Supplementary Information

Below is the link to the electronic supplementary material.

Supplementary file1 (MP4 35472 KB)
